# Beyond the Pseudogene: p17/PERMIT as a Mitochondrial Trafficking Protein Linking Aging and Neurodegeneration

**DOI:** 10.1111/acel.70175

**Published:** 2025-07-16

**Authors:** Onder Albayram, Natalia Oleinik, Besim Ogretmen

**Affiliations:** ^1^ Department of Pathology and Laboratory Medicine, ER‐AL Neurovascular Protection Laboratory Medical University of South Carolina Charleston South Carolina USA; ^2^ Ralph H. Jackson Department of Veterans Affairs Medical Center Charleston South Carolina USA; ^3^ Department of Neuroscience Medical University of South Carolina Charleston South Carolina USA; ^4^ Department of Biochemistry and Molecular Biology Medical University of South Carolina Charleston South Carolina USA; ^5^ Hollings Cancer Center Medical University of South Carolina Charleston South Carolina USA

## Abstract

The misclassification of functional genomic loci as pseudogenes has long obscured critical regulators of cellular homeostasis, particularly in aging‐related pathways. One such locus, originally annotated as RPL29P31, encodes a 17‐kDa protein now redefined as PERMIT (Protein that Mediates ER–Mitochondria Trafficking). Through rigorous experimental validation—including antibody development, gene editing, lipidomics, and translational models—p17/PERMIT has emerged as a previously unrecognized mitochondrial trafficking chaperone. Under aging or injury‐induced stress, p17 mediates the ER‐to‐mitochondria translocation of Ceramide Synthase 1 (CerS1), facilitating localized C18‐ceramide synthesis and autophagosome recruitment to initiate mitophagy. Loss of p17 impairs mitochondrial quality control, accelerating neurodegeneration, and sensorimotor decline in both injury and aging models. This Perspective highlights p17 as a paradigm‐shifting discovery at the intersection of lipid signaling, mitochondrial biology, and genome reannotation, and calls for a broader reassessment of the “noncoding” genome in aging research. We summarize a rigorous multi‐platform validation pipeline—including gene editing, antibody generation, lipidomics, proteomics, and functional rescue assays—that reclassified p17 as a bona fide mitochondrial trafficking protein. Positioned at the intersection of lipid metabolism, organelle dynamics, and genome reannotation, p17 exemplifies a growing class of overlooked proteins emerging from loci historically labeled as pseudogenes, urging a systematic reevaluation of the “noncoding” genome in aging research.

## Graphical abstract

This Perspective highlights the experimental validation of p17/PERMIT, a mitochondrial trafficking protein encoded by a locus previously annotated as a pseudogene. The work outlines a generalizable framework for uncovering functional proteins hidden in the noncoding genome, with implications for mitochondrial quality control and aging‐related neurodegeneration.

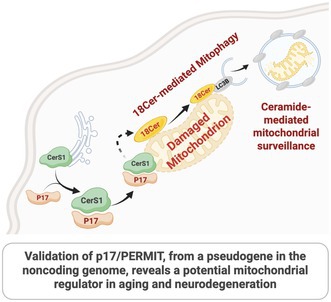

## 1 Uncovering Proteins Hidden in Plain Sight

Over the past decade, a critical realization has emerged: many genomic loci previously annotated as pseudogenes may, in fact, encode biologically active proteins with essential functions. This error in classification—often based solely on sequence homology or presumed lack of transcription—has obscured key regulators of cell biology, particularly in stress and aging contexts. A striking case is p17, now reclassified as PERMIT (Protein that Mediates ER–Mitochondria Trafficking), a 17‐kDa protein once dismissed as a ribosomal pseudogene (Oleinik et al. 2019). Recent discoveries have repositioned p17 as a critical mediator of ceramide‐directed mitophagy, a lipid‐driven quality control mechanism with broad implications for brain aging, injury, and mitochondrial resilience (Karakaya et al. 2024; Oleinik et al. 2023).

The case of p17/PERMIT offers more than correction—it represents a rare and important discovery: a functional protein emerging from a locus once presumed biologically irrelevant. Unlike many other pseudogenes that regulate gene expression through noncoding RNA functions, p17 produces a functional protein that actively governs a core process in cellular homeostasis—mitochondrial quality control through lipid trafficking. This reclassification not only redefines the locus itself but opens new dimensions in mitochondrial cell biology by introducing a previously unrecognized mitochondrial carrier mechanism.

While p17/PERMIT is the central focus of this Perspective, our goal is not to recount a singular discovery, but to use this well‐validated example to illustrate a broader conceptual shift: that a subset of genomic loci previously classified as pseudogenes may encode biologically active, stress‐inducible proteins. This perspective builds on reclassifications of loci such as PTENP1, which acts as a competing endogenous RNA regulating tumor suppressor pathways (Poliseno et al. 2010); NANOGP8, which encodes a protein promoting stemness in cancer cells (Zhang et al. 2006); and SLC22A10, an orphan transporter family member that has been reclassified as a lipid carrier (Yee et al. 2024). Beyond these examples, additional pseudogenes, such as MYLKP1 (Han et al. 2011), CYP4Z2P (Li et al. 2017; Zheng et al. 2015), and KRASP1 (Poliseno et al. 2010), have been shown to encode functional proteins that contribute to muscle contractility, tumor growth, and modulation of MAPK signaling, respectively. Each of these reclassifications encountered significant skepticism before gaining broader acceptance, reflecting ongoing debates over how to define protein‐coding potential. These cases collectively underscore the limitations of homology‐based genome annotation and highlight the biological diversity of reclassified pseudogenes. In this context, p17/PERMIT represents not an isolated anomaly, but the latest addition to a growing class of functionally validated proteins emerging from loci that have long been dismissed as genomic relics.

## 2 From Annotation to Function: The Case of p17/PERMIT

We position p17 not only as a case study, but also as a biological metaphor for how deeply entrenched annotation paradigms can obscure the existence of functional proteins. Initially annotated as RPL29P31 due to partial homology with the ribosomal protein L29, p17 was long presumed non‐functional (Law et al. 1996). This presumption has proven incorrect. Through a series of rigorous validations—including peptide‐specific antibody generation, siRNA and CRISPR knockdowns, gain‐of‐function rescue assays, peptide competition studies, co‐immunoprecipitation, and mass spectrometry—p17 was shown to be a biologically active, non‐ribosomal protein (Oleinik et al. 2019). These validations were strengthened by epitope‐targeting mutagenesis, mitochondrial lipidomics, and cross‐reactivity exclusion using commercial RPL29 antibodies. Each of these platforms, used orthogonally and across species, contributed to the unequivocal recognition of p17/PERMIT as a genuine, functional protein that mediates mitochondrial quality control in response to stress.

What distinguishes p17 is not merely its reclassification, but its mechanistic novelty. Unlike classical mitochondrial transporters, p17 operates as a chaperone‐based carrier, transiently activated under stress or injury conditions. Its role bridges two previously unconnected systems: lipid biosynthesis at the ER and mitophagy initiation at the mitochondria. As such, p17 emerges as a new type of mitochondrial mediator—one whose activity is conditional, lipid‐specific, and functionally engaged during aging and disease states.

## 3 Functional Identity: p17 as a Lipid Trafficking Regulator

p17 serves as a trafficking chaperone that links cellular stress to mitochondrial turnover. Under normal conditions, p17 is sequestered at mitochondria‐associated ER membranes (MAMs) via binding to Drp1. Upon cellular stress, S‐nitrosylation of Drp1 triggers the release of p17, allowing it to bind Ceramide Synthase 1 (CerS1) and direct its translocation to the outer mitochondrial membrane. There, CerS1 produces C18‐ceramide, a lipid signal that recruits LC3‐positive autophagosomes to initiate mitophagy. This pathway represents a lipid‐mediated, protein‐guided mechanism for mitochondrial quality control—distinct from canonical PINK1/Parkin signaling (Oleinik et al. 2019, 2023).

The introduction of this ER‐to‐mitochondria lipid transport axis via p17 adds a new layer of regulation to mitochondrial maintenance. While the role of ceramides in apoptosis and metabolic disease has been studied, the concept that specific lipid species such as C18‐ceramide are actively generated within mitochondria to signal mitophagy is novel. It suggests that p17 acts not just as a carrier, but as a regulatory gatekeeper for mitochondrial lipid composition and turnover.

## 4 Aging and Neurodegeneration: Translational Validation in the Brain

The importance of p17 extends beyond cell lines and into complex physiology. In p17 knockout (p17KO) mouse models, loss of function leads to impaired mitochondrial clearance, accumulation of dysfunctional organelles, and progressive neurological deficits. In *PNAS Nexus* (2024) (Karakaya et al. 2024), p17KO mice subjected to repetitive mild brain injury exhibited reduced C18‐ceramide synthesis, defective mitophagy, and long‐term cognitive impairments. In *Aging Cell* (2023) (Oleinik et al. 2023), aged p17KO mice displayed Purkinje cell degeneration, mitochondrial structural abnormalities, and behavioral phenotypes resembling age‐related motor decline. These converging lines of evidence support p17 as a vital component of the aging brain's resilience machinery.

Moreover, human postmortem analyses demonstrate concordant decreases in p17, CerS1, and mitochondrial LC3 lipidation in brain regions vulnerable to degeneration, reinforcing the translational bridge. The pathway uncovered here is not simply stress‐responsive—it is an intrinsic, endogenous defense mechanism against mitochondrial deterioration, whose failure accelerates aging phenotypes.

## 5 Rethinking the “Noncoding” Genome in Aging Research

The story of p17 is not an isolated case—it is a paradigm shift. Functional proteomic reevaluation has already reclassified pseudogene‐encoded proteins like PTENP1 (Poliseno et al. 2010), NANOGP8 (Zhang et al. 2006), and others (Yee et al. 2024; Wang et al. 2010). p17 adds to this emerging class, demonstrating that high‐resolution, experimental approaches—such as lipidomics, antibody engineering, and stress‐model systems—can uncover latent regulatory biology missed by sequence‐based annotation. Aging research in particular must integrate this perspective: the genome's “dark matter” may hold keys to longevity, repair, and resilience.

Beyond genome reannotation, the example of p17 calls for a shift in how we interpret stress response pathways. Mitochondrial maintenance is not governed solely by canonical signaling proteins or organelle‐localized enzymes. Instead, dynamic, stress‐inducible proteins like p17—encoded by previously overlooked loci—may serve as key nodes in the network that determines cellular fate across the aging trajectory.

## 6 The Hidden Proteome of Aging: A Call to Action

The discovery of p17/PERMIT demands a reevaluation of our assumptions about genomic annotation. More importantly, it presents a tractable model for targeting mitochondrial dysfunction in aging and injury. By validating its function through orthogonal platforms and extending its relevance into neurodegeneration, we assert that p17 is not merely a correction in nomenclature—it is a new node in the biological network governing healthy aging. We encourage the aging research community to look again at the pseudogene landscape, now not with dismissal, but with precision and curiosity. Aging is the sum of accumulated stress and compromised repair. The validation of p17/PERMIT exemplifies how careful molecular characterization—especially of loci previously dismissed as pseudogenes—can reveal new mechanisms that preserve mitochondrial integrity and promote cellular resilience. The proteome of aging is still incomplete; p17 reminds us that some of its most important regulators may still be hidden in plain sight.

The experimental pipeline outlined in this Perspective—from antibody generation and gene editing to lipidomics and proteomics—provides a roadmap for reclaiming overlooked functional proteins from the pseudogene landscape. Future applications of such cross‐platform validation strategies will be essential to expand the functional proteome and uncover new therapeutic targets within the presumed ‘noncoding’ genome.

While the story of p17/PERMIT is grounded in a single locus, the experimental validation framework that revealed its function is broadly applicable. From in silico reannotation and antibody generation to CRISPR gene editing, lipidomics, proteomics, and translational modeling, each step contributed to uncovering a bona fide protein hidden in plain sight. This Perspective offers not just a molecular insight, but a methodological roadmap: a replicable approach to reclaiming overlooked functional proteins from the pseudogene landscape. As the aging field advances, applying such cross‐platform validation pipelines will be essential to expand the functional proteome and unlock new therapeutic targets encoded within the presumed “noncoding” genome.

## Author Contributions

O.A., N.O., and B.O. have written the perspective.

## Conflicts of Interest

The authors declare no conflicts of interest.

## Data Availability

Data sharing is not applicable to this article as no new data were created or analyzed in this study.
